# Diverse roles of the *CIPK* gene family in transcription regulation and various biotic and abiotic stresses: A literature review and bibliometric study

**DOI:** 10.3389/fgene.2022.1041078

**Published:** 2022-11-15

**Authors:** Chen Yang, Jin Yi-feng, Wang Yushu, Gao Yansong, Wang Qi, You Xue

**Affiliations:** ^1^ College of Life Science, Agriculture and Forestry, Qiqihar University, Qiqihar, China; ^2^ Heilongjiang Provincial Key Laboratory Resistance Gene Engineering, Qiqihar, China

**Keywords:** *CIPK*, gene family, biotic and abiotic stresses, bibliometric, web of science, protein kinases, transcription regulation

## Abstract

CIPKs are a subclass of serine/threonine (Ser/Thr) protein kinases. CBLs are ubiquitous Ca^2+^ sensors that interact with CIPK with the aid of secondary Ca^2+^ messengers for regulation of growth and development and response to stresses faced by plants. The divergent roles of the CIPK-CBL interaction in plants include responding to environmental stresses (salt, cold, drought, pH, ABA signaling, and ion toxicity), ion homeostasis (K^+^, NH_4_^+^, NO_3_^−^, and microelement homeostasis), biotic stress, and plant development. Each member of this gene family produces distinct proteins that help plants adapt to diverse stresses or stimuli by interacting with calcium ion signals. CIPK consists of two structural domains—an N-terminal domain and a C-terminal domain—connected by a junction domain. The N-terminal domain, the site of phosphorylation, is also called the activation domain and kinase domain. The C-terminal, also known as the regulatory domain of CIPK, further comprises NAF/FISL and PPI. CBL comprises four EF domains and conserved PFPF motifs and is the site of binding with the NAF/FISL domain of CIPK to form a CBL-CIPK complex. In addition, we also performed a bibliometric analysis of the *CIPK* gene family of data extracted from the WoSCC. A total of 95 documents were retrieved, which had been published by 47 sources. The production over time was zigzagged. The top key terms were *gene*, *CIPK*, *abiotic stress*, and *gene expression*. *Beijing Forestry University* was the top affiliation, while *The Plant Cell* was the top source. The genomics and metabolomics of this gene family require more study.

## 1 The *CIPK* gene family

CIPKs are a subclass of serine/threonine (Ser/Thr) protein kinases. CBL (calcineurin B-like proteins) are ubiquitous Ca^2+^ sensors that interact with CIPK (CBL-interacting protein kinases) with the aid of secondary messengers Ca^2+^ to regulate growth and development, and also respond to various stresses faced by plants (Sanyal et al., 2016). Ca^2+^ plays a regulatory role in the formation of the CBL-CIPK complex and the enhancement of kinase domain activity (Sánchez-Barrena et al., 2007). The various roles of CIPK-CBL interaction in plants include responding to environmental stress (salt stress, cold stress, drought stress, pH stress, ABA signaling, ion toxicity), ion homeostasis (K^+^ homeostasis, NH_4_^+^ homeostasis, NO_3_^−^ homeostasis, microelement homeostasis), biotic stress, and plant development (Ding et al., 2022). Each member of this gene family produces distinct proteins that help plants to adapt to diverse stresses or stimuli by interacting with calcium ion signals.

### 1.1 Structure of the CIPK-CBL complex

**FIGURE 1:**
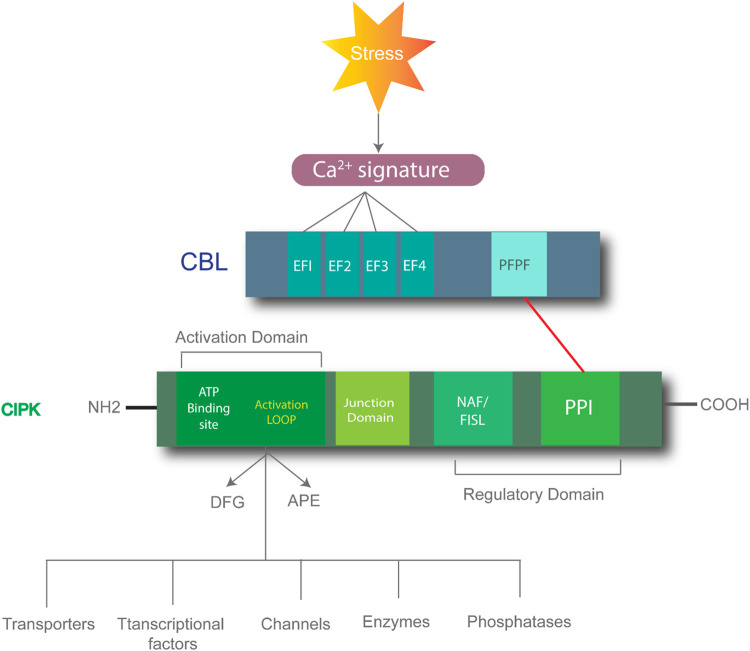
Process of CBL-CIPK complex-mediated responses. Stress can induce the calcium ion signatures which bind to EF1-EF4 domains on the CBL. CIPK consists of the N-terminal domain comprising the activation domain and the C-terminal domain comprising the regulatory domain, which includes the NAF/FISL and PPI domains. The red line shows the CBL and CIPK interaction. This complex formation regulates downstream processes.

CIPK consists of two structural domains: an N-terminal domain and a C-terminal domain, which are connected by a junction domain. The N-terminal domain, the site of phosphorylation, is also called the activation domain and kinase domain (Guo et al., 2001). This domain comprises three conserved amino acids (Ser, Thr, and Tyr) that are crucial for the proper CIPK functioning and activity. CIPK activity is inhibited if any one of the three amino acids is mutated to Asn. The C-terminal is also the regulatory domain of CIPK and further comprises NAF/FISL and PPI (Sanyal et al., 2015). CBL includes four EF domains and conserved PFPF motifs and is the site of binding with the NAF/FISL domain of CIPK to form the CBL-CIPK complex. When the intracellular level of Ca^2+^ is less, the NAF domain is not bound to the PFPF domain due to the self-inhibitory property of FISL the domain, thus blocking the kinase activity (Batistic et al., 2008). When the Ca^2+^ level increases, the PFPF motifs of CBL bind to the NAF domain of CIPK due to the conformational change in protein structure and hydrophobic interactions, causing a release of kinase activity and downstream regulation for various stress responses (Figure 1) (Sánchez-Barrena et al., 2007).

### 1.2 How many genes?

Each plant has a different number of *CIPK* genes that have been discovered so far. For instance, the total number of *CIPK* genes in *Arabidopsis, Populus,* and *Cassava* are presented in Supplementary Table S1. There are several *CBL* and *CIPK* genes found in various plants, some examples are mentioned in Tables 1, 2. The *CIPK* gene family is further divided into two groups: Those with more introns and those with fewer introns. Additionally, fluorescent labeling reveals that the majority of CIPK proteins are found in the cytoplasm and nucleus. In the cytoplasm and nucleus, the GFP fusion proteins of several CIPKs, including CIPK1, CIPK2, CIPK3, CIPK4, CIPK7, CIPK8, CIPK10, CIPK14, CIPK17, CIPK21, CIPK23, and CIPK24, exhibit significant fluorescence (Mao et al., 2016b). However, the subcellular localization and targeting mechanisms of most CBL-CIPK complexes are yet mysterious.

**TABLE 1:** List of plants with identified *CIPK* and *CBL* genes.

Botanical name	Family	Common name	CIPKs	CBLs	References
*Citrus sinensis*	*Rutaceae*	Sweet orange	7 *CsCIPK*	8 *CsCBL*	Shu et al. (2020)
*Setaria italica*	*Poaceae*	Millet	35 *SiCIPK*	—	Zhao et al. (2019)
*Solanum commersonii*	*Solanaceae*	Nightshade	26 *ScCIPK*	10 *ScCBL*	Esposito et al. (2019)
Malus domestica	*Rosaceae*	Apple	34 *MdCIPK*	—	Niu et al. (2018)
*Medicago sativa*	*Fabaceae*	Alfalfa	58 *MsCIPK*	23 *MsCBL*	Du et al. (2021)
*Beta vulgaris*	*Amaranthaceae*	Beet	20 *BvCIPK*	—	Wu et al. (2022)
*Arabidopsis thaliana*	*Brassicaceae*	Rockcress	26 *AtCIPK*	10 *AtCBL*	Kolukisaoglu et al. (2004)
*Oryza sativa*	*Poaceae*	Rice	33 *OsCIPK*	10 *OsCBL*	Kanwar et al. (2014)
*Vitis vinifera*	*Vitaceae*	Grapes	20 *VvCIPK*	8 *VvCBL*	Xi et al. (2017)
*Capsicum annuum*	*Solanaceae*	pepper	26 *CaCIPK*	9 *CaCBL*	Ma et al. (2019a)
*Chenopodium quinoa*	*Amaranthaceae*	Quechua	41 *CqCIPK*	16 *CqCBL*	Xiaolin et al. (2022)
*Pyrus bretschneideri*	*Rosaceae*	Pear	28 *PbCIPK*	—	Tang et al. (2016)
*Carya illinoinensis*	*Juglandaceae*	Hardy pecan	30 *CiCIPK*	9 *CiCBL*	Zhu et al. (2022)
*Dendrobium catenatum*	*Orchidaceae*	Dendrobium	24 *DcaCIPK*	—	Wan et al. (2019)
*Brassica napus*	*Brassicaceae*	Canola	23 *BnaCIPK*	7 *BnaCBL*	Zhang et al. (2014)
*Cicer arietinum*	*Fabaceae*	Chickpea	*22 CaCIPK*	—	Poddar et al. (2021)
*Glycine max*	*Fabaceae*	Soya bean	52 *CIPK*	—	Zhu et al. (2016)
*Solanum melongena*	*Solanaceae*	Egg Plant	26 *SmCIPK*	10 *SmCIPK*	Li et al. (2016)
*Sorghum bicolor*	*Poaceae*	Broomcorn	32 *SbCIPK*	—	Guo et al. (2015)
*Manihot esculenta*	*Euphorbiaceae*	Cassava	26 *MeCIPK*	10 *MeCBL*	Mo et al. (2018)
*Prunus mume*	*Rosaceae*	Plum	16 *PmCIPK*	—	Li et al. (2019)
*Camellia sinensis*	*Theaceae*	Tea plant	18 *CsCIPK*	7 *CsCBL*	Wang et al. (2020)
*Lonicera japonica*	*Caprifoliaceae*	Honeysuckle	17 *LjCIPK*	6 *LjCBL*	Huang et al. (2021)
*Zea mays*	*Poaceae*	Maize	43 *ZmCIPK*	—	Chen et al. (2011)
*Gossypium raimondii*	*Malvaceae*	Cotton plant	41 *GrCIPK*	—	Wang et al. (2016)
*Gossypium arboreum*	*Malvaceae*	Cotton plant	18 *GaCIPK*	—	Wang et al. (2016)

**TABLE 2:** *CIPK* genes and their function in *Arabidopsis thaliana*.

Gene	Function	Reference
*AtCIPK1*	Encodes a protein kinase that interacts with CBL, ECT1 and ECT2	Lu et al. (2020)
*AtCIPK2*	Act as a calcium sensor that is involved in growth and development, Negative regulator of the responses to cold	Kudla et al. (1999)
*AtCIPK3*	Encodes a serine-threonine protein kinase	Ju et al. (2022)
*AtCIPK4*	CBL-interacting protein kinase 4; autophosphorylation activity	Nemoto et al. (2011)
*AtCBL4*	Known as Arabidopsis SOS3 and contribute to salt stress by activating the Na^+^/H^+^ antiporter	Ye et al. (2013)
*AtSOS1*	It maintains Na^+^ at low level	Wang et al. (2019)
*AtCIPK7*	It responds to cold tolerance	Huang et al. (2011)
*AtCBL9*	Forms a complex with AtCIPK23 to regulate potassium homeostasis under low potassium stress	Lara et al. (2020)
*AtCIPK24*	SOS2 gene is required for intracellular Na^+^ and K^+^ homeostasis	Liu et al. (2000)

### 1.3 Brief introduction to some members of the *CIPK* gene family

A total of 26 *CIPK* genes have been identified in *Arabidopsis* (Kolukisaoglu). Several of these genes and their roles are described in Table 3.

**TABLE 3:** *CIPK* roles in transcriptional and post-transcriptional regulation in different plants.

Role	References
Post-transcriptional modification of CIPKs, was identified in plant signaling pathways and abiotic resistance	Xian et al. (2020)
*CIPKs* plays diverse roles in phosphorylation in cold stress	Reviewed by (Barrero-Gil and Salinas, 2013)
The CIPKs may be involved in post-transcriptional modification of the development of the rhizomes and the construction of defense systems of the plants	Yang (2022)
CIPK-CBL complex raises cytosolic free Ca^2+^ levels, enhancing CIPK kinase activity and triggering a phosphorylation cascade	Batistič and Kudla (2009)
In Arabidopsis, the AKT1 channel is phosphorylated by CIPK23, which enhances the K^+^ uptake activity of AKT1 under K^+^-deficient conditions	(Li et al., 2006; Xu et al., 2006)
CIPKs interact with PP2Cs by binding the kinase domain of CIPK and the protein phosphatase interacting motif (PPI) in the regulatory domain	Lan et al. (2011)

Individual *CIPK* genes from other plant species have also been reported and characterized in terms of function. For instance, *OsCIPK23* overexpression improved rice drought tolerance by increasing the expression levels of drought-related genes (Yang et al., 2008). Drought activated the cotton *CIPK* gene *GhCIPK6*, the overexpression of which in *Arabidopsis* improved drought tolerance (He et al., 2013). These findings imply that the *CIPK* family plays a significant role in the development of plant stress tolerance.

### 1.4 Roles of *CIPK* in transcriptional regulation in plants

The divergent roles of the CIPK-CBL interaction in plants include response to environmental stress (salt stress, cold stress, drought stress, pH stress, ABA signaling, ion toxicity), ion homeostasis (K^+^, NH_4_^+^, NO_3_^−^, and microelement homeostasis), biotic stress, and plant development (Ding et al., 2022). We discuss these roles in the following sections.

#### 1.4.1 Environmental stress: salt stress

**FIGURE 2:**
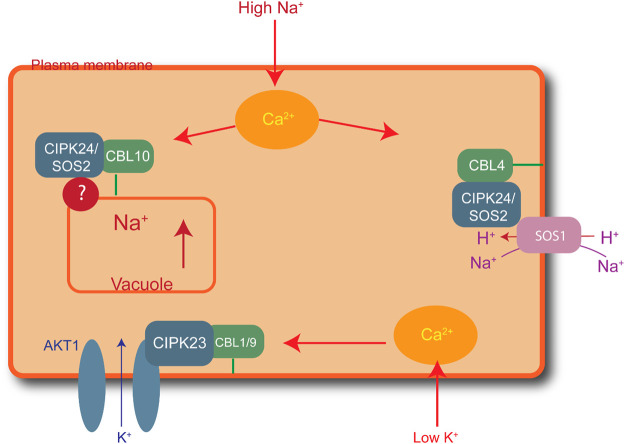
Role of CIPK-CBL complexes in managing salt stress in *Arabidopsis*. CBL4-CIPK24 (SOS2) activates SOS1 for the extracellular transfer of excess sodium ions. Similarly, CBL10-CIPK24 (SOS2) transfers extra sodium ions inside the vacuole, and CBL1/9-CIPK23 plays a role in maintaining a balance of potassium ions by activating AKT1 receptors in the plasma membrane to import potassium ions in low-potassium conditions.

Elevated salt levels in plants can cause salt toxicity, resulting in halted growth and development. Similarly, extremely low cellular K^+^ levels can affect plant development. Plants have developed protection mechanisms against salt stress. For instance, to lessen increasing cytoplasmic levels of Na^+^, plants overcome the threat of salt damage by increasing Na^+^ discharge, decreasing Na^+^ intake, and sequestering Na^+^ into the vacuoles. The first identified CBL-CIPK complex to maintain ion homeostasis is SOS (salt overly sensitive) pathway. High Na^+^ concentration activates the formation of CBL4/SOS3 and CIPK24/SOS2 complex due to increased concentration of Ca^2+^ (Yin et al., 2020). This complex activated SOS1, is a plasma membrane anti-porter for Na^+^/H^+^ ions. SOS1 helps plants maintain the ion balance by transporting Na^+^ from the cytoplasm to the extracellular space and controls Na^+^ transport from root to shoot. CBL10-CIPK24/SOS2 maintains Na^+^ levels by vacuole sequestration (Shi et al., 2000; Yin et al., 2020). Similarly, CBL1/9-CIPK23 plays a role in maintaining the K^+^ balance in response to low cellular levels. Low levels of intracellular K^+^ activate Ca^2+^, resulting in the formation of the CBL1/9-CIPK23 complex, in which AKTI is located in the plasma membrane, as shown in Figure 2 (Cheong et al., 2007). The CIPK23 kinase domains activate the AKTI channel in the plasma membrane by physically interacting and phosphorylating the channel to initiate K^+^ uptake into the cytoplasm (Li et al., 2006).

#### 1.4.2 Environmental stress: cold stress

**FIGURE 3:**
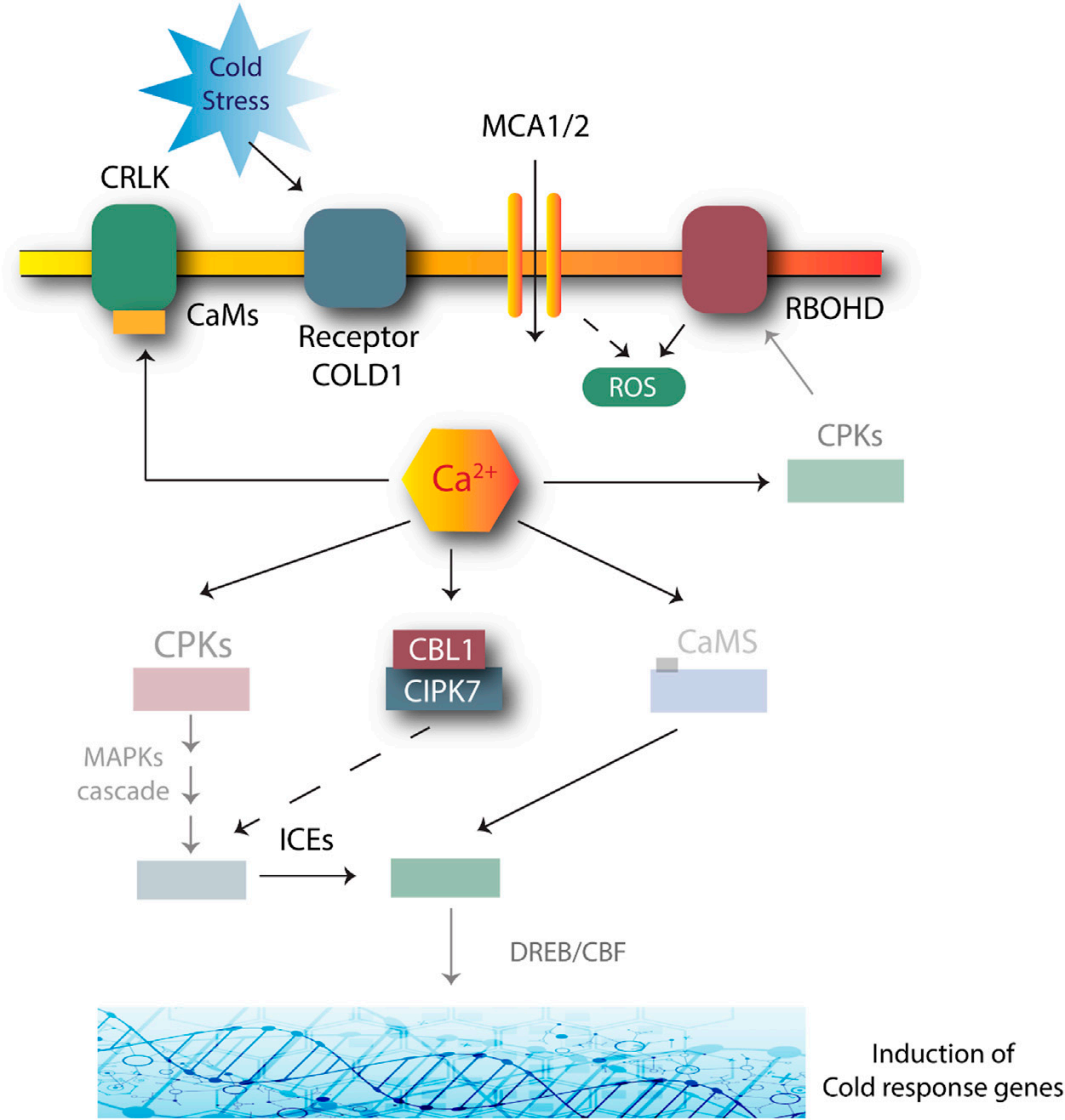
Role of CIPK-CBL complexes in managing cold stress in *Arabidopsis.* Many genes are involved in this response. Cold stress is first perceived by COLD1 receptors. The signals activate the secondary Ca^2+^ messengers. The transient changes produced in a cell due to Ca^2+^ activation and AtSRC2 produce ROS due to the NADPH oxidase activity of the AtRBOHF. Increased ROS production positively regulates the Ca^2+^ channels. CBL1-CIPK plays a role by phosphorylating and activating the MAPK cascades. CRLK1 or CRLK2 interacts with calmodulin (CaM) to further activate the MAPK cascade. The MAPK pathway suppresses the breakdown of ICE1 to tolerate stress. Dashed lines: unclear mechanisms; solid lines: induction.

Cold stress can have severe effects on overall plant survival. Cold stress can inhibit plant growth as it reduces membrane fluidity and affects water and nutrient absorption; thus disturbing cellular metabolism and reactions (Chinnusamy et al., 2007). Plants have developed mechanisms to respond to decreasing temperature conditions through various pathways, including the CIPK7-CBL1 complex-mediated approach (Shinozaki et al., 2003). CIPK7-CBL1 may be involved in the response to cold stress by producing sugars, as sugars reduce the risk of cell damage by cold. AtCIPK7 may lead to the phosphorylation of sucrose synthase for sucrose metabolism, as shown in Figure 3 (Huang et al., 2011).

#### 1.4.3 Environmental stress: drought stress

Drought stress can seriously threaten plant growth through reduced efficiency water use, smaller leaf size, stem elongation, etc. CBL1/9-CIPK23 responds to drought stress in plants by altering the sensitivity of ABA in guard cells. CIPK11 negatively regulates the stress response in coordination with transcriptional factor Di19-3 (Ma X. et al., 2019; Ma Y. et al., 2019).

#### 1.4.4 Environmental stress: ABA signaling

ABA is a vital phytohormone in plants that plays diverse roles, including stomata opening and closing, pathogen defense, seed germination, and plant growth and development. Studies have revealed the role of the CIPK-CBL complex in the regulation of the signal transduction pathway. CIPK3/CBL9 and CIPK15/CBL1 play vital roles in ABA signaling. Experiments have revealed a negative regulatory role of AtCBL9 in ABA signaling during seed germination. A study in plants lacking CBL9 with mutant cbl9 showed the accumulation of higher levels of abscisic acid and hypersensitivity to abscisic acid (Pandey et al., 2004; Pandey et al., 2008). Similarly, to understand the involvement of CIPK15/CBL1 in ABA signaling, plants with mutant CBL1 (cbl1) have been studied. Study results showed that CBL1 is not involved in the regulation of ABA signaling while the interaction of CIPK1 with CBL1 and CBL9 plays a role in the mediation of abscisic acid signaling (D'Angelo et al., 2006). Similarly, CIPK6 loss-of-function lines (cipk6) also showed a higher accumulation of abscisic acid in seedlings, thus demonstrating the role of CIPK6 in ABA signaling (Chen et al., 2013).

#### 1.4.5 Environmental stress: ion toxicity

Cadmium ions can accumulate in plants and are highly toxic to plant growth and development (Cuypers et al., 2010). AtCPK11 plays a role in overcoming cadmium stress by enhancing expression of the ABA signaling pathway (Gu et al., 2021). IRT1 is cadmium transported in roots and the ABA pathway reduces the two crucial transcriptional factors, FIT and bHL039, which regulate IRT1 to block the entry of cadmium and other ions (Fan et al., 2014). Similarly, CIPK23 causes IRT1 degradation to stop the entry of toxic ions into cells (Dubeaux et al., 2018).

#### 1.4.6 Environmental stress: pH stress

**FIGURE 4:**
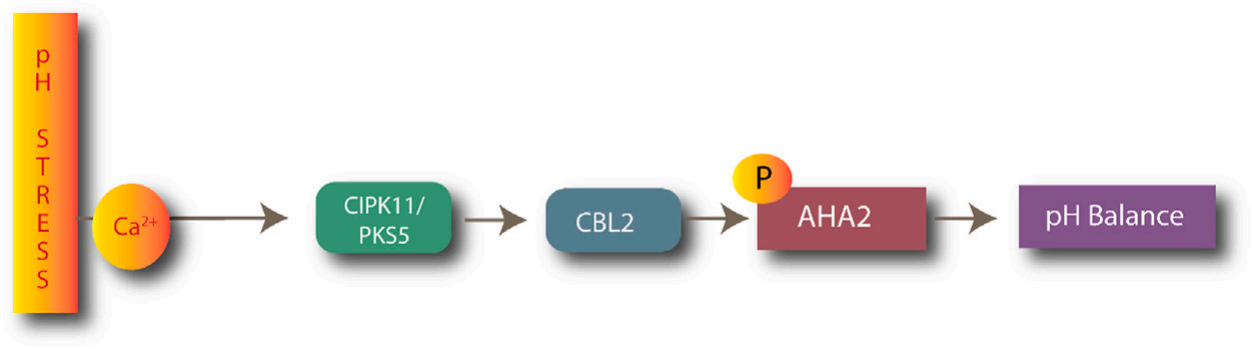
Process of maintaining pH balance by CBL2-CIPK11 complexes after activation of AHA ATPases.

CIPK11/CBL2 plays a key role in pH stress management in plants. CIPK11 targets the H^+^-ATPases AHA2, which is involved in extracellular acidification. CIPK11 (PKS5) negatively regulates H^+^-ATPase AHA2, as shown in Figure 4 (Fuglsang et al., 2007). The AtCIPK11-AtCBL2 complex phosphorylates H^+^-ATPase AHA2, preventing it from interacting with the 14-3-3 protein in alkaline conditions (Sanyal et al., 2020).

#### 1.4.7 Ion homeostasis

Ion homeostasis is a dynamic process for maintaining the physiological processes of plants. The CIPK-CBL complexes play crucial roles in the homeostasis of NO_3_−, NH^4+^, K^+^, and microelements (Mulet et al., 2020). The CBL-CIPK complex allows plant cells to sequester extra Mg^2+^ into vacuoles, thereby protecting plant cells from high Mg toxicity (Tang et al., 2015). CBL4-CIPK5 complex confers salt, but not drought and chilling, tolerance by regulating ion homeostasis (Huang et al., 2020).

#### 1.4.8 NO_3_^–^ homeostasis

Nitrate is the chief source of nitrogen for plants and acts as a signal molecule by inducing the expression of nitrate-associated genes, a process called the primary nitrate response. Nitrate induction includes two responses; namely, the high-affinity (km = 30 µM) and low-affinity (km = 0.9 µM) (Liu et al., 2020) stages. The genes of the *CIPK* family, including *CIPK8* and *CIPK23*, play a crucial role in nitrate homeostasis by regulating the low and high-affinity stages of nitrate induction, respectively. *CIPK8* positively regulates the low-affinity response by inducing the expression of the *CHL1* (*NRT1*.1) and *NRT2*.1 transporters. CIPK23 is a negative regulator of high-affinity response and acts in antagonism with CIPK8 (Zhao et al., 2018). The CBL1/0-CIPK23 complex is located in the plasma membrane, where CIPK23 can convert low-affinity CHL1 to high-affinity by phosphorylation of threonine residues in low-nitrate conditions. Thus, at low nitrate concentrations, higher primary nitrate reactions are prevented. In contrast, high nitrate concentrations inhibit CHL1 phosphorylation by binding to low-affinity sites. This produces high primary nitrate reactions and, ultimately, transports nitrates (Ho et al., 2009).

#### 1.4.9 NH_4_^+^ homeostasis

**FIGURE 5:**
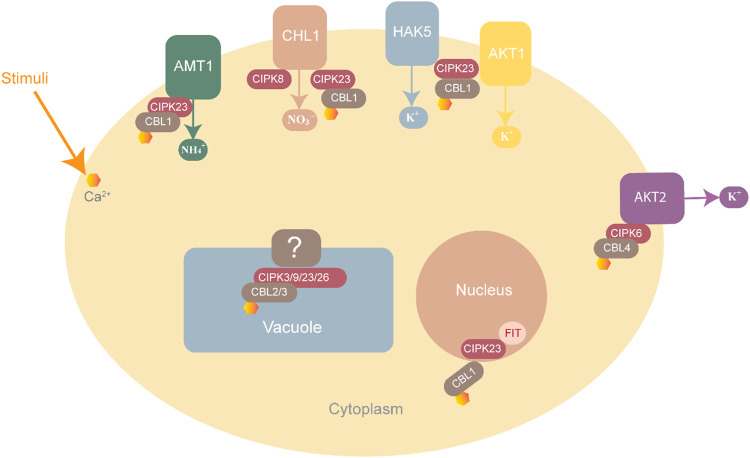
CBL-CIPK complexes involved in ion homeostasis. NH_4_^+^ homeostasis is maintained through AMT1 inactivation by the CBL-CIPK23 complex to block entry into the cell. K^+^ homeostasis is maintained by three complexes, CBL1-CIPK23, which interact with HAK5 and AKT1 while CBL4-CIPK6 interacts with AKT2 for K^+^ uptake into the cell. NO_3_ homeostasis is maintained by CIPK8 and CIPK23, which interact with CHL1 as positive and negative regulators respectively.

High levels of NH_4_^+^ are toxic for plant growth. The CBL1-CIPK23 complex helps maintain ammonium balance by phosphorylating and inactivating the threonine residue of cytosolic AMT1, thus preventing the entry of further ammonium ions (Figure 5) (Straub et al., 2017).

#### 1.4.10 K^+^ homeostasis

**FIGURE 6:**
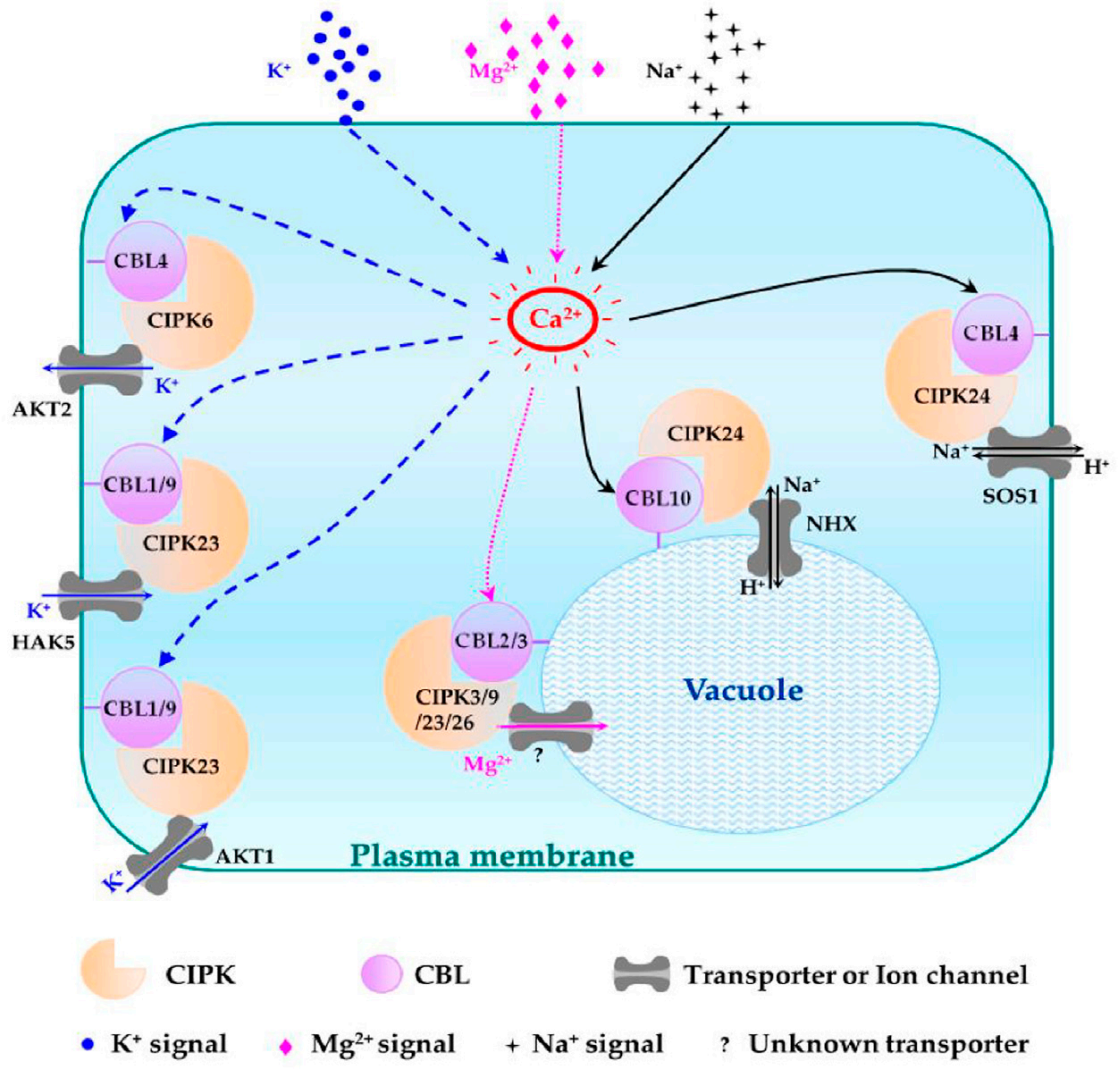
Schematic representation of different CBL-CIPK complexes and their functions in regulating Na^+^, K^+^, and Mg^2+^ homeostasis. The regulatory pathways of K^+^, Mg^2+^, and Na^+^ are indicated in blue, pink, and black lines, respectively. Question mark (?): unknown tonoplast-localized transporter (Mao et al., 2016a).

Plants usually suffer at low potassium concentrations. The CBL1/9-CIPK23-AIP1-AKT1 pathway plays a role in maintaining a balance of this ion in plants. CBL1/9 recruits CIPK23 to form an activating complex at the root cells, where CIPK23 activates the AKT1 transporter to increase potassium ion intake by phosphorylating AKT1 (Srivastava et al., 2020). Other than AKT1, CIPK23 can also regulate the potassium ion concentration by interacting with HAK5 (K^+^ transporter) and KUP4 (K^+^ regulator) (Srivastava et al., 2020). Many essential microelements in homeostasis are also maintained by CBL-CIPK families. For instance, CBL1/9-CIPK11 and CBL1/9-CIPK23, CIPK7, and CIPK21 play roles in iron homeostasis, as shown in Figure 6 (Ding et al., 2022).

#### 1.4.11 Biotic stress

**FIGURE 7:**
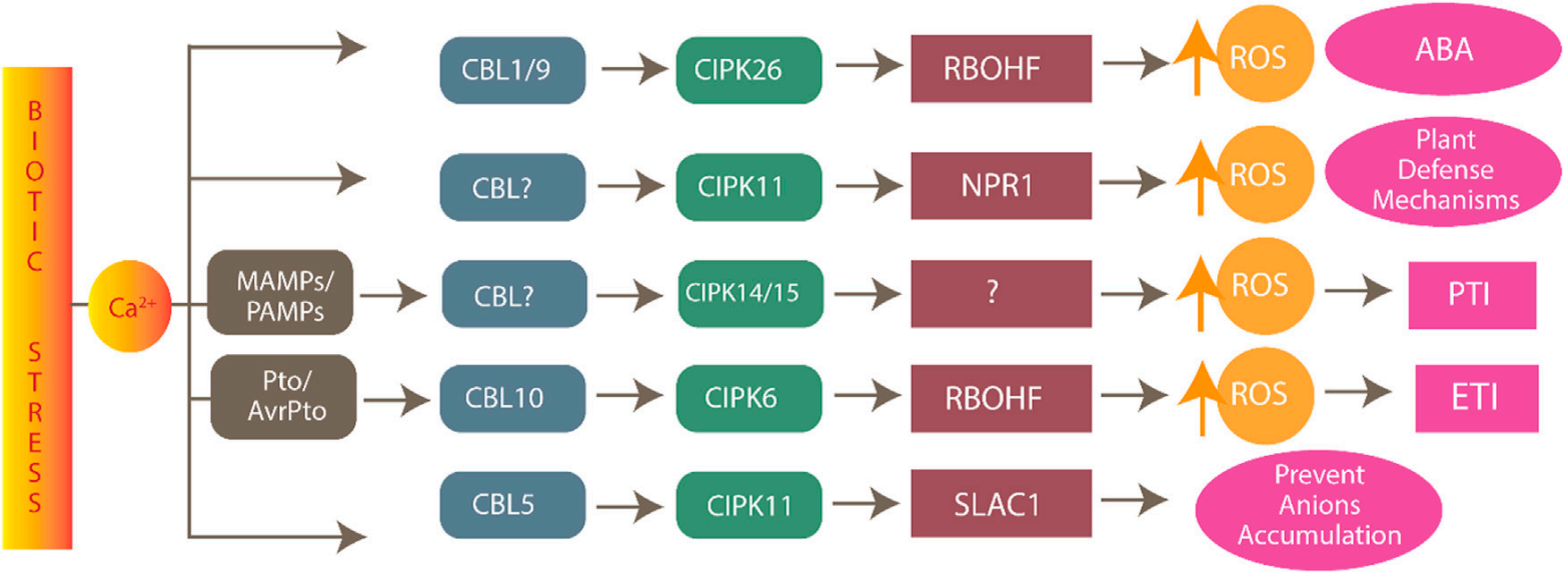
CBL-CIPK complexes involved in response to biotic stress in plants.

CIPK plays a vital role in protecting plants from biotic stress such as pathogens by directly or indirectly increasing reactive oxygen species (ROS) production (Torres, 2010). ROS are produced by plants through variable defense mechanisms (e.g., PAMP-triggered immunity (PTI) and effector-triggered immunity (ETI)) as a sign that the plant has recognized the pathogen and started to protect itself from biotic stress (Torres, 2010). For instance, CIPK26 protects plants from pathogens *via* the production of ROS. Similarly, respiratory burst oxidase homolog (RBOH) is also involved in ROS production in plants. The CBL1/9-CIPK26 complex activates RBOH, which further triggers ROS production *via* the ABA pathway and leads to stomata closure (Fuglsang et al., 2007). Similarly, CBL5-CIPK11 activates other plant defense mechanisms by activating NPR1 (nonexpressor of pathogenesis-related genes), which acts downstream of salicylic acid, which is well known for defense in plants and activates defense genes. The CBL5-CIPK11 complex also reportedly regulates WRKY38 and WRKY62 in disease response (Figure 7) (Tena et al., 2011).

#### 1.4.12 Plant development

CIPK6/12/13/19 plays a vital role in plant development and growth. CIPK12 and CIPK19 are key regulators in the growth of pollen tube tips in plants (Zhou et al., 2015). CIPK13 plays a part in reversible protein phosphorylation, which is essential for maintaining many plant processes such as metabolism, photosynthesis, gene expression, cell development, and structure formation (Schliebner et al., 2008; Schönberg and Baginsky, 2012). CIPK6 is involved in cell division, enhancement of auxin sensitivity, and root growth and development (Tripathi et al., 2009). Finally, CIPK25 aids in the regulation of root meristem development and size (Tripathi et al., 2009).

### 1.5 CBL-CIPK complex mediates different signaling pathways in plants

As explained above, the CBL-CIPK complex is involved in responses to high salinity, osmotic or drought stress, cold, pH, ABA, K^+^, nitrate, and other stresses. Crosstalk occurs between the CBL-CIPK complex and other classical pathways such as the CDPK, AMP-activated protein kinase (AMPK), salt overly sensitive (SOS), and reactive oxygen species (ROS) pathways. AtCBL4/SOS3 was the first CBL protein identified in *Arabidopsis* and was shown to function specifically in the SOS pathway, interacting with AtCIPK24/SOS2 under salinity stress in roots (Liu and Zhu, 1998; Qiu et al., 2002). During germination and seedling development, AtCBL1 reacts to signals involving glucose and gibberellin (GA) (Li et al., 2013). AtCBL1 and AtCBL9 regulate the *Arabidopsis* NADPH oxidase RbohF after interacting with AtCIPK26 in the ROS pathway (Kimura et al., 2013).

## 2 Bibliometric analysis

Bibliometrics is the application of statistical techniques to evaluate the scientific content of books, papers, and other publications (Ellegaard and Wallin, 2015). In library and information science, bibliometric techniques are preferably employed. Bibliometrics examines and analyzes scientific metrics and different indicators highly associated with the respective domains (Ellegaard and Wallin, 2015). The earliest bibliometrics studies date back to the late 19th century and changed significantly following the Second World War in the wake of the “periodical crises” and the new technological possibilities provided by computing tools (Wolfram, 2003). The Science Citation Index resulting from Eugene Garfield and Derek John de Solla Price’s citation network analysis became the cornerstone of organized bibliometrics research programs in the early 1960s (Wolfram, 2003). Creating the citation graph, which displays a network of citations between texts, is the foundation of the widely used bibliometric technique known as citation analysis (Hutchins et al., 2019). To examine the influence of their area bibliometric methodologies have been applied to specific publications to identify especially influential papers in certain fields of study (Weingart, 2005). The creation of thesauri, evaluating reader usage, and descriptive linguistics are approaches frequently incorporated in bibliometrics.

### 2.1 Data sources for bibliometric analysis

Currently, bibliometric analysis can be performed using several databases. However, the development of Scopus and the Web of Science has simplified citation analysis. With certain restrictions, data sources including Dimensions, Google Scholar, and PubMed are also used for data extraction (AlRyalat et al., 2019; Donthu et al., 2021). In 2010, new initiatives in support of open citation data challenged the background of infrastructures for citation data. For example, Web of Science and Scopus have dramatically eased meta-data analysis (Ellegaard, 2018). The Web of Science database provides users access to billions of cited sources in the humanities, social sciences, and life sciences dating back to 1900. This database can be accessed through the website: https://www.webofscience.com. Millions of entries from journals, books, and conference proceedings are included in the Scopus extended abstract and citation database. Scopus can be accessed *via* the website: https://www.scopus.com/home.uri.

#### 2.1.1 Web of Science

The Web of Science (WoS) is a subscription platform available to institutions and offers access to numerous databases containing reference and citation data from conference proceedings, academic journals, and other publications in a variety of disciplines (AlRyalat et al., 2019). WoS was created by the “Institute for Scientific Information” but is currently held by Clarivate, formerly Thomson Reuters’ Intellectual Property and Science division (Wikipedia, 2022).

#### 2.1.2 Scopus

Elsevier is among the top abstract and citation databases. Scopus was first introduced in 2004 and comprises almost 36,377 titles from approximately 11,678 publishers worldwide (Scopus, 2022). Among these titles, 34,346 are peer-reviewed journals in top-tier subject areas (biological sciences, physical sciences, social sciences, and health sciences). It encompasses certain different sources, for instance, trade journals, book series, and journals (Khudhair et al., 2020). Each year, different parameters such as the h-Index, CiteScore, SCImago Journal Rank, and SNIP numerical quality measures are used to evaluate each journal included in the Scopus database to ensure that it is of a high enough standard (Source Normalized Impact per Paper) (Rankings, 2022). Patent database searches are also incorporated into Scopus searches (Kulkarni et al., 2009).

### 2.2 Introduction to bibliometric analysis tools

Although several bibliometric databases are available, each was created with a specific objective (Mallig, 2010; Choudhri et al., 2015). Among bibliometric analysis tools, Bibliometrix has the most comprehensive collection of methods and is appropriate for practitioners because of Biblioshiny (Aria and Cuccurullo, 2017). VOSviewer can import and export data from a variety of sources and offers excellent visualization (Van Eck and Waltman, 2017). SciMAT provides the most robust pre-processing and export capabilities (Cobo et al., 2012) and has been used in biomedical sciences (Granada et al., 2020), applied intelligence, and machine learning (Rincon-Patino et al., 2018; López-Robles et al., 2021). Due to the variety of characteristics, researchers must decide on the desired analytical results and select the choice that best serves their objectives.

#### 2.2.1 VOSviewer

VOSviewer (visualization of similarities) is a program for creating and visualizing bibliometric networks. Different types of networks can be developed using VOSviewer. For instance, bibliographic coupling and co-authorship relationships can be shown as attractive networks (Van Eck and Waltman, 2010). VOSviewer allows the detailed analysis of bibliometric maps from various aspects and provides the viewer with the ability to zoom and scroll through the maps to perform detailed examination. To prevent the overlapping of labels, smart labeling algorithms are used. For viewing a moderately large number of items in bibliometric maps, the viewing capability of VOSviewer is remarkable compared to other software (Van Eck and Waltman, 2010). Similarly, VOSviewer can also be used to produce citation and co-citation networks. These networks can be journals, researchers, or individual articles (Van Eck and Waltman, 2010). Data retrieved from the Web of Science, Scopus, Dimensions, Lens, and PubMed databases can be used to create co-authorship, citation-based, and co-occurrence networks (VOSviewer, 2022). Similarly, data downloaded from the APIs of Crossref, Europe PMC, and OpenAlex can also be used to create networks. This program can save screenshots of data as bitmaps and vectors with high resolution (VOSviewer, 2022).

#### 2.2.2 Bibliometrix

All of the primary bibliometric methods of analysis are included in the open-source, scientometric, and bibliometric research tool known as Bibliometrix developed by Aria and Cuccurullo (2017). No coding experience is required to use this software to analyze fields like journals, topics, authors, timespan, etc. (Bibliometrix, 2022). The Bibliometrix package includes a variety of features to extract bibliographic data from databases. The best examples of these databases include SCOPUS, WoS, PubMed, Clarivate Analytics, Digital Science, and Dimensions. Bibliometrics can create data matrices for coupling, scientific collaboration analysis, co-citation, and co-word analysis. It can also be used for structural analysis of new information in data and developments.

#### 2.2.3 Gephi

Gephi is another free program for bibliometric data visualization as massive network graphs (Bastian et al., 2009). To speed exploration, Gephi utilizes a 3-D platform to display graphs in real-time (Gephi, 2021).

## 3 Bibliometric analyses of the *CIPK* gene family

A bibliometric analysis of the *CIPK* gene family was performed. Data were extracted from the Web of Science Core Collection on August 26, 2022. We selected three editions of the core collection; namely, the “Science Citation Index Expanded (SCI-EXPANDED)-1999-present”, “Conference Proceedings Citation Index (AHCI)—2003-present”, and “Emerging Sources Citation Index (ESCI0—2017-present”. The keyword strings *CIPK* OR *CIPK* GENE OR *CIPK* GENE FAMILY were used. Publications were collected from 2003 to 2022. A total of 95 articles were retrieved from the database.

### 3.1 Information

The main information is provided in Table 4. The time duration was 2003 to 2022. A total of 95 documents published by 47 sources were retrieved.

**TABLE 4:** Main information about the data.

Description	Results
Timespan	2003:2022
Sources (Journals, Books, etc.)	47
Documents	95
Annual growth rate %	12.88
Document average age	5.61
Average citations per doc	34.72
References	2,844
Document contents	
Keywords plus (ID)	309
Author’s keywords (DE)	274
Authors	
Authors	507
Authors of single-authored docs	1
Authors collaboration	
Single-authored docs	1
Co-authors per Doc	6.35
International co-authorships %	16.84
Document types	
Article	87
Meeting abstract	1
Review	7

### 3.2 Publication analysis of the *CIPK* gene family

**FIGURE 8:**
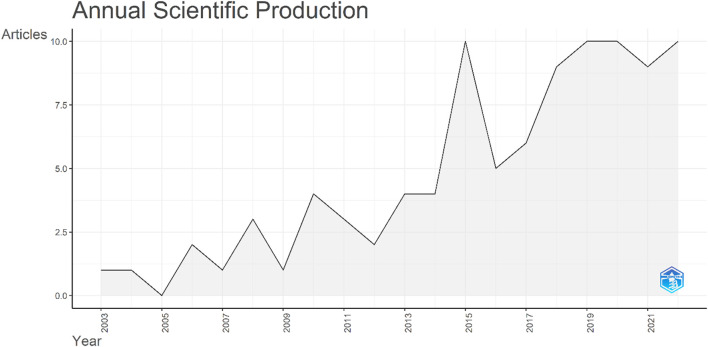
Total number of publications on the CIPK gene family over the study period. The *x*-axis indicates the years while the *y*-axis indicates the number of publications.

The raw data were analyzed to extract the publications per annum. The data were first published in 2003, with a single article, entitled *Isolation and characterization of a novel rice Ca* ^*2+*^ *-regulated protein kinase gene involved in responses to diverse signals including cold, light, cytokinins, sugars and salts*. The production over time zigzagged and no papers were published in 2005. In 2015, the number of publications reached double figures, with a total of 10 articles published. However, the number decreased to five articles in 2016. Since then, a continuous increase was observed (Figure 8).

### 3.3 Relevancy network

**FIGURE 9:**
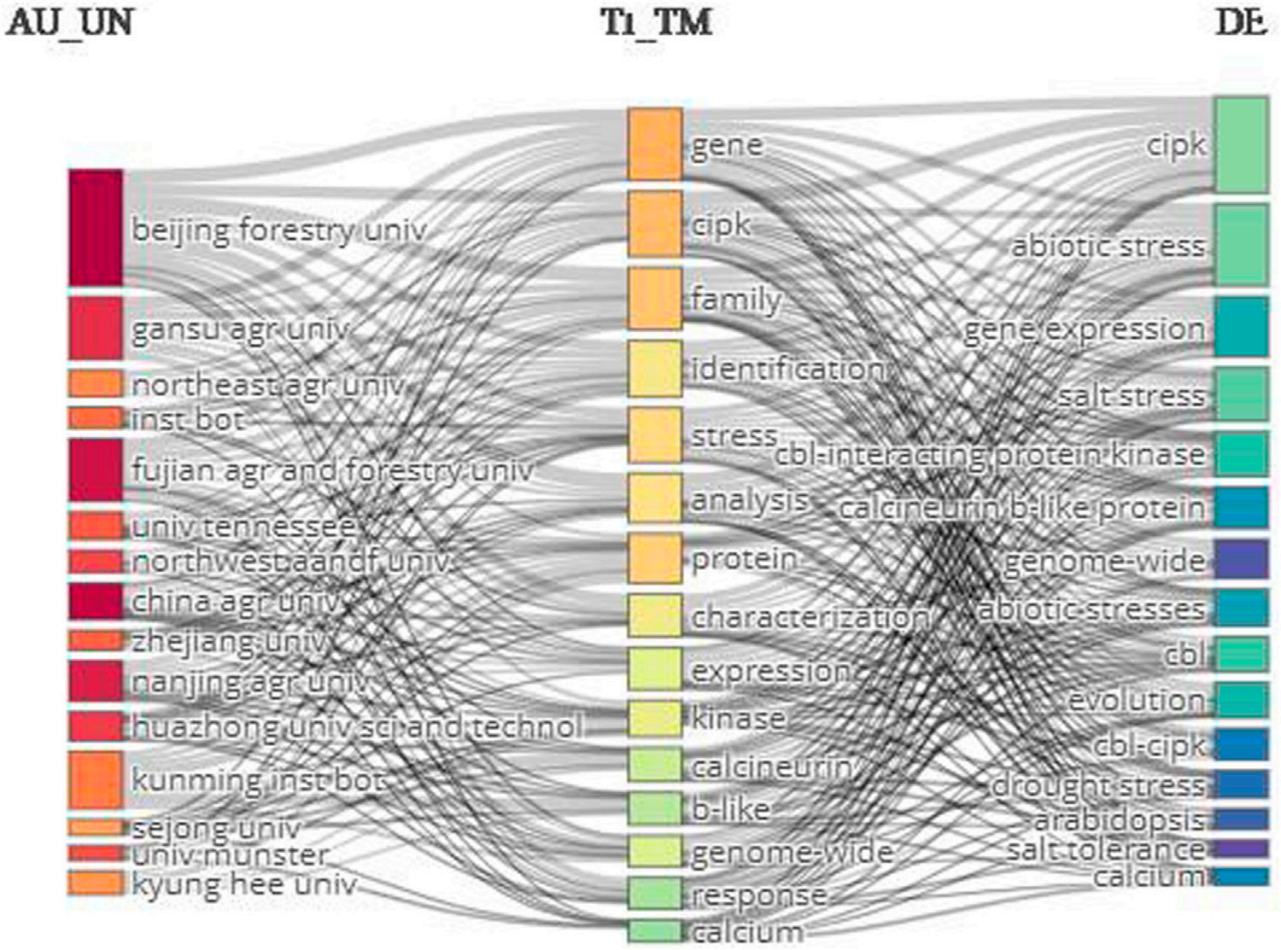
Three-field network map. The right-hand, middle, and left-hand sides show the keywords, terms extracted from titles, and author affiliations, respectively.

The relevancy network was mapped by targeting 1) authors’ affiliations, 2) title terms, and 3) keywords. The top title terms were gene, cipk, family, and identification. The leading keywords were cipk, abiotic stress, gene expression, and salt stress. The top affiliation was Beijing Forestry University (Figure 9).

### 3.4 Most relevant sources

**FIGURE 10:**
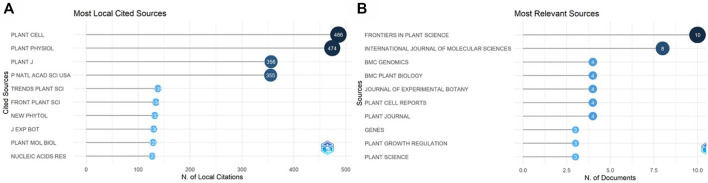
Most relevant sources. **(A)** The most locally cited sources. **(B)** The most relevant sources according to the number of publications.

The retrieved data was analyzed to find the most relevant sources (Figure 10A). *Frontiers in Plant Science* was the most common journal, with 10 publications. The current IF of the journal is 6.627 and is in category Q1. The most locally cited sources were also identified (Figure 10B), among which *Plant Cell* was first with 486 citations, followed by *Plant Physiology Journal*, with 474 citations.

### 3.5 Authors-citation network analysis

**FIGURE 11:**
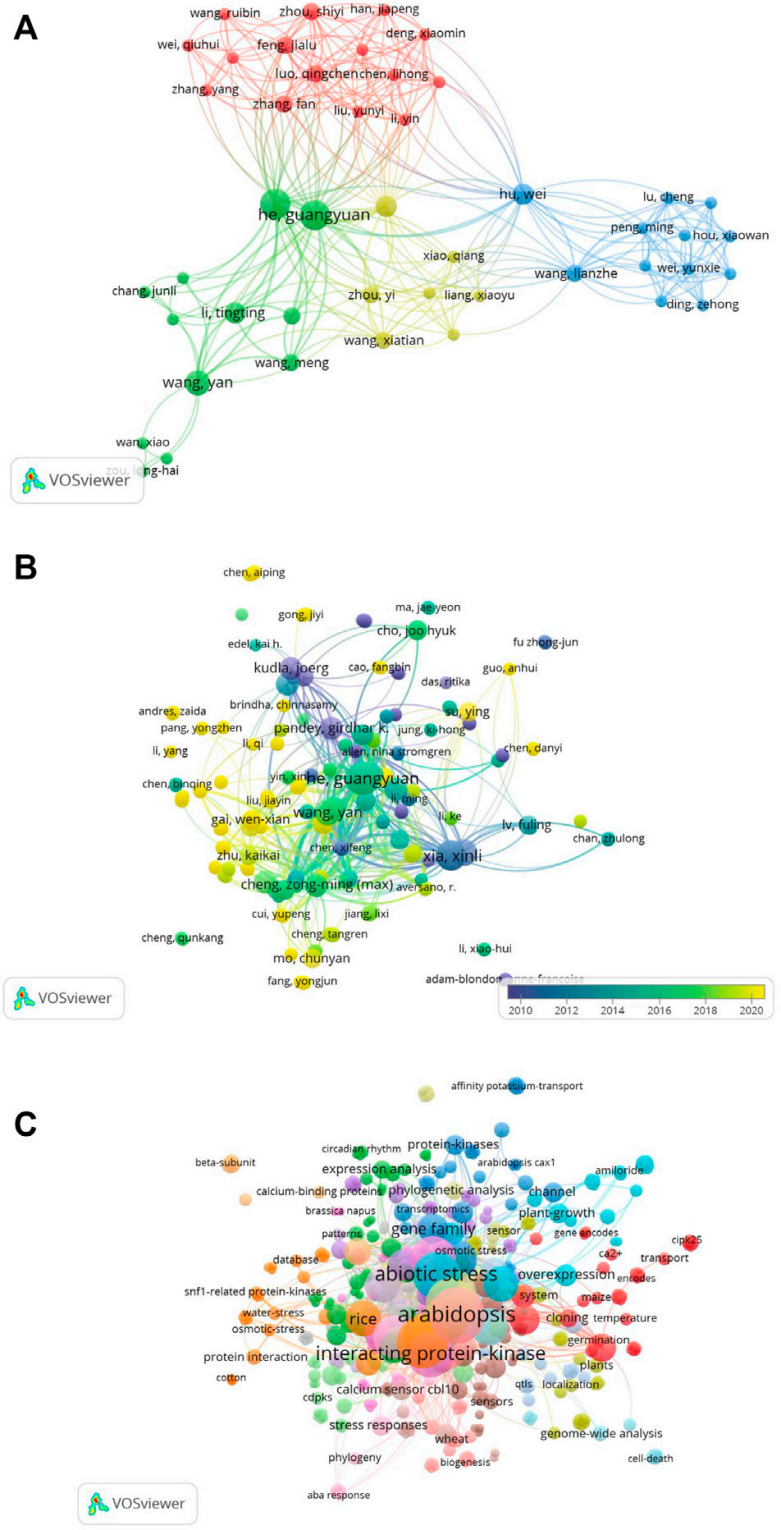
**(A)** Co-authors to authors’ network. Only linked authors are shown. VOSviewer was used to draw the network. **(B)** Citations to author network map including a total of 532 authors. The overlay visualization of the linked author network is shown. **(C)** Co-occurrence of keywords. A total of 538 keywords were identified and mapped using VOSviewer. Abiotic stress, abscisic acid, and *Arabidopsis* were the leading terms.

We used VOSviewer to map the co-authorship-to-author network (Figure 11A). Similarly, we mapped the network of citations to the authors. A total of 532 authors were included in these publications. We mapped only the linked author network, as shown in Figure 11B. Analysis of the co-occurrence of keywords identified a total of 538 keywords, among which abiotic stress, abscisic acid, and *Arabidopsis* were the leading terms (Figure 11C).

### 3.6 Term co-occurrence map

**FIGURE 12:**
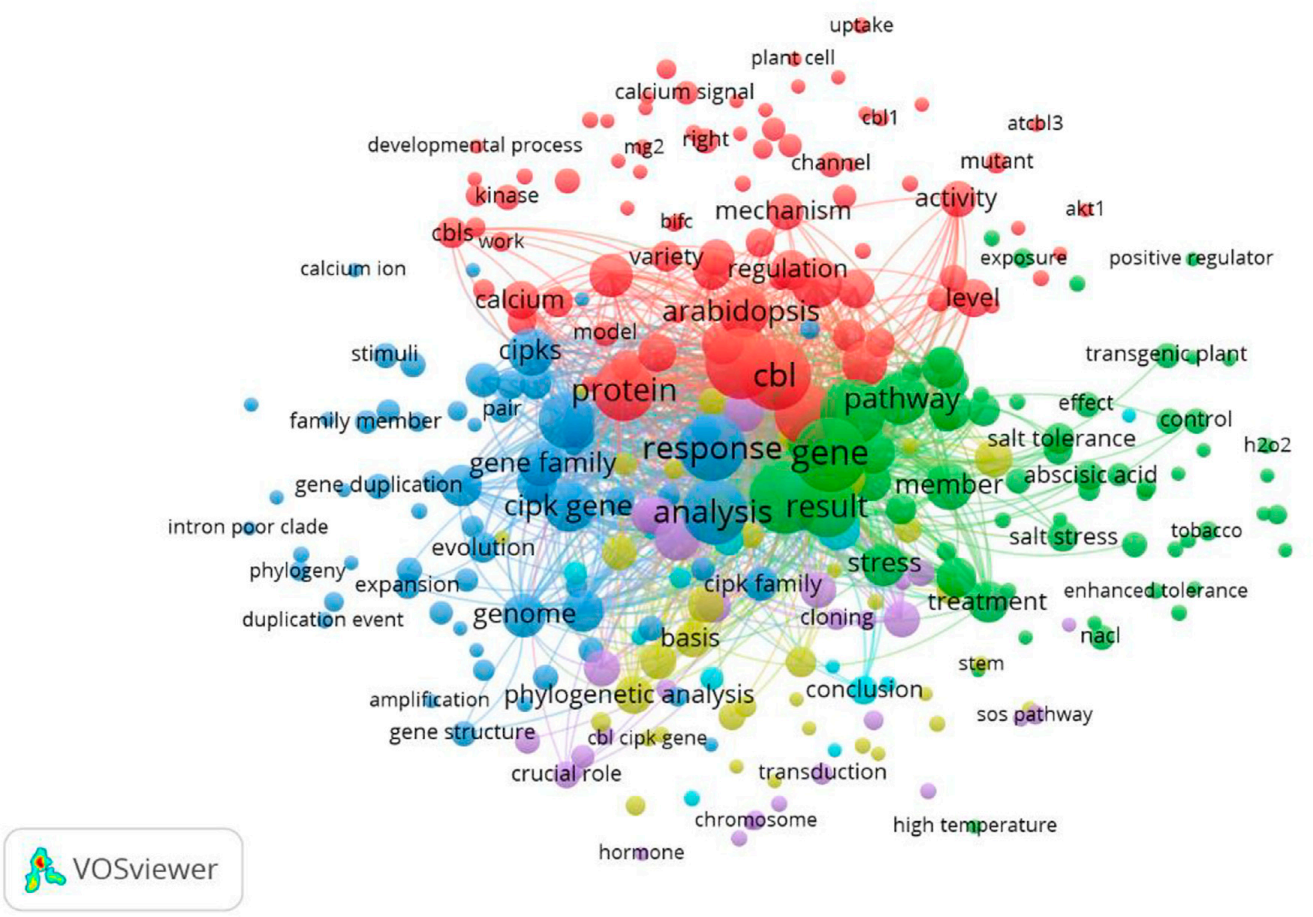
Term co-occurrence map. A total of 2,856 terms were extracted from the titles and abstracts. The mapped network is sketched using a minimum occurrence of 3. A total of 300 terms were mapped.

We used VOSviewer to sketch the term co-occurrence map based on the text data. The terms were extracted from the titles and abstracts. A total of 2,856 terms were included. To draw the network map, we set the threshold criterion of minimum occurrence to 3 for key terms and applied “binary” counting. A total of 300 terms met this criterion. The mapped network is shown in Figure 12.

## 4 What should be discovered in the future?

CIPK proteins play important roles in the Ca^2+^ signaling pathway and affect plant development, in addition to participating in biotic and abiotic stress responses. CIPKs have been identified and functionally characterized in many crops such as *Arabidopsis*, rice, maize, and canola (Niu et al., 2018), but less is known about CIPKs in wheat and other economically important crops. Much remains to be studied in this gene family in terms of genomics and metabolomics analysis.
