# Supplementary material for: Diverse roles of the CIPK gene family in transcription regulation and various biotic and abiotic stresses: A literature review and bibliometric study
Source: Front Genet. 2022 Nov 15;13:1041078. doi: 10.3389/fgene.2022.1041078 (PMC9705351; doi:10.3389/fgene.2022.1041078)
Supplement: Supplementary file 1 [file Table1.DOCX]

**Supplementary file S_1.**

**Table 1.1: Total number of genes of CIPK family in different plant species**

| Arabidopsis |  | Populus |  | Cassava |  |
| --- | --- | --- | --- | --- | --- |
| Gene name | **Gene ID** | **Gene name** | **Gene ID** | **Gene name** | **Gene ID** |
| AtCIPK1 | *At3g17510* | PtCIPK1 | *DQ997692* | MeCIPK1 | *KP675718* |
| AtCIPK2 | *At5g07070* | PtCIPK2 | *DQ997693* | MeCIPK2 | *KP675719* |
| AtCIPK3 | *At2g26980* | PtCIPK3 | *DQ997694* | MeCIPK3 | *KP675720* |
| AtCIPK4 | *At4g14580* | PtCIPK4 | *DQ997695* | MeCIPK4 | *KP675721* |
| AtCIPK5 | *At5g10930* | PtCIPK5 | *DQ997696* | MeCIPK5 | *KP675722* |
| AtCIPK6 | *At4g30960* | PtCIPK6 | *DQ997717* | MeCIPK6 | *KP675723* |
| AtCIPK7 | *At3g23000* | PtCIPK7 | *DQ997697* | MeCIPK7 | *KP675724* |
| AtCIPK8 | *At4g24400* | PtCIPK8 | *DQ997698* | MeCIPK8 | *KP675725* |
| AtCIPK9 | *At1g01140* | PtCIPK9 | *DQ997699* | MeCIPK9 | *KP675726* |
| AtCIPK10 | *At5g58380* | PtCIPK10 | *DQ997700* | MeCIPK10 | *KP675727* |
| AtCIPK11 | *At2g30360* | PtCIPK11 | *DQ997701* | MeCIPK11 | *KP675728* |
| AtCIPK12 | *At4g18700* | PtCIPK12 | *DQ997702* | MeCIPK12 | *KP675729* |
| AtCIPK13 | *At2g34180* | PtCIPK13 | *DQ997703* | MeCIPK13 | *KP675730* |
| AtCIPK14 | *At5g01820* | PtCIPK14 | *DQ997704* | MeCIPK14 | *KP675731* |
| AtCIPK15 | *At5g01810* | PtCIPK15 | *DQ997705* | MeCIPK15 | *KP675732* |
| AtCIPK16 | *At2g25090* | PtCIPK16 | *DQ997706* | MeCIPK16 | *KP675733* |
| AtCIPK17 | *At1g48260* | PtCIPK17 | *DQ997707* | MeCIPK17 | *KP675734* |
| AtCIPK18 | *At1g29230* | PtCIPK18 | *DQ997708* | MeCIPK18 | *KP675735* |
| AtCIPK19 | *At5g45810* | PtCIPK19 | *DQ997709* | MeCIPK19 | *KP675736* |
| AtCIPK20 | *At5g45820* | PtCIPK20 | *DQ997718* | MeCIPK20 | *KP675737* |
| AtCIPK21 | *At5g57630* | PtCIPK21 | *DQ997710* | MeCIPK21 | *KP675738* |
| AtCIPK22 | *At2g38490* | PtCIPK22 | *DQ997711* | MeCIPK22 | *KP675739* |
| AtCIPK23 | *At1g30270* | PtCIPK23 | *DQ997712* | MeCIPK23 | *KP675740* |
| AtCIPK24 | *At5g35410* | PtCIPK24 | *DQ997713* | MeCIPK24 | *KP675741* |
| AtCIPK25 | *At5g25110* | PtCIPK25 | *DQ997714* | MeCIPK25 | *KP675742* |
| AtCIPK26 | *At5g21326* | PtCIPK26 | *DQ997715* |  |  |
|  |  | PtCIPK27 | *DQ997716* |  |  |
